# Retinal ganglion cell analysis in multiple evanescent white dot syndrome

**DOI:** 10.1186/1471-2415-14-132

**Published:** 2014-11-19

**Authors:** Hideo Akiyama, Hirotaka Itakura, Danjie Li, Tomoyuki Kashima, Keisuke Nitta, Yukitoshi Shimoda, Ryo Mukai, Shoji Kishi

**Affiliations:** Department of Ophthalmology, Gunma University Graduate School of Medicine, 3-39-15 Showa-machi, Maebashi, Gunma 371-8511 Japan

**Keywords:** Optical coherence tomography, Ganglion cell analysis, MEWDS

## Abstract

**Background:**

Multiple evanescent white dot syndrome (MEWDS) is an acute and usually unilateral retinopathy that occurs predominantly in young adults. This report presents the outcomes of ganglion cell analysis (GCA) in MEWDS.

**Case presentation:**

A 41-year-old woman was diagnosed as MEWDS in right eye. At her initial visit, the deviation map of the ganglion cell analysis showed there was a decrease of the ganglion cell layer (GCL) + inner plexiform layer (IPL) thickness in both eyes, even though her left eye was not affected. A 29-year-old woman was also diagnosed as MEWDS in right eye. Although the deviation map of ganglion cell analysis showed there was a decrease of the GCL + IPL thickness in both eyes at her initial visit, her right eye was not affected.

**Conclusion:**

GCA indicated there was a decrease (<1% of the distribution of normals) of the ganglion cell layer + inner plexiform layer thickness in both the affected and fellow eyes in 7 of 9 patients diagnosed as MEWDS in our hospital. Although the lesions responsible for MEWDS are thought to disrupt the photoreceptor outer segments, we observed changes in the inner retina in both the affected and fellow eye of MEWDS patients.

## Background

Multiple evanescent white dot syndrome (MEWDS), which Jampol et al. first reported in 1984, is an acute and usually unilateral retinopathy that occurs predominantly in young adults [1]. The main complaints of MEWDS patients include blurred vision, photopsia, or visual field defect [2]. MEWDS has been considered an inflammatory disease with characteristic yellowish spots at the level of the retinal pigment epithelium or deep retina. Using optical coherence tomography (OCT), Li et al. demonstrated that the lesions responsible for MEWDS appear to disrupt the photoreceptor outer segments [3]. Although the clinical features of MEWDS have been well described, the definitive cause of this disease remains unknown.

Recent advances in OCT technology have enabled more detailed and precise quantitative assessments of glaucoma structural changes [4]. In addition, implementation of the Cirrus high definition (HD)-OCT (Carl Zeiss Meditec, Dublin, CA) ganglion cell analysis (GCA) algorithm has made it possible to detect and measure the thickness of the macular ganglion cell-inner plexiform layer (GCIPL) with excellent of reproducibility [5–8]. Mwanza et al. demonstrated that the ability of the macular GCIPL parameters to discriminate between normal eyes and eyes with early glaucoma was high and comparable to that of the best peripapillary retinal nerve fiber layer and optic nerve head parameters [7].

In the current report, we found that the deviation map of GCA showed there was a decrease (<1% of distribution of normal) in the ganglion cell layer (GCL) + inner plexiform layer (IPL) thickness in both the affected and fellow eyes in 7 of 9 patients. Although the lesions responsible for MEWDS are thought to disrupt the photoreceptor outer segments, we speculate that MEWDS patients might exhibit changes in the inner retina in both the affected and fellow eye.

## Case presentation

### Case 1

**Figure 1:**
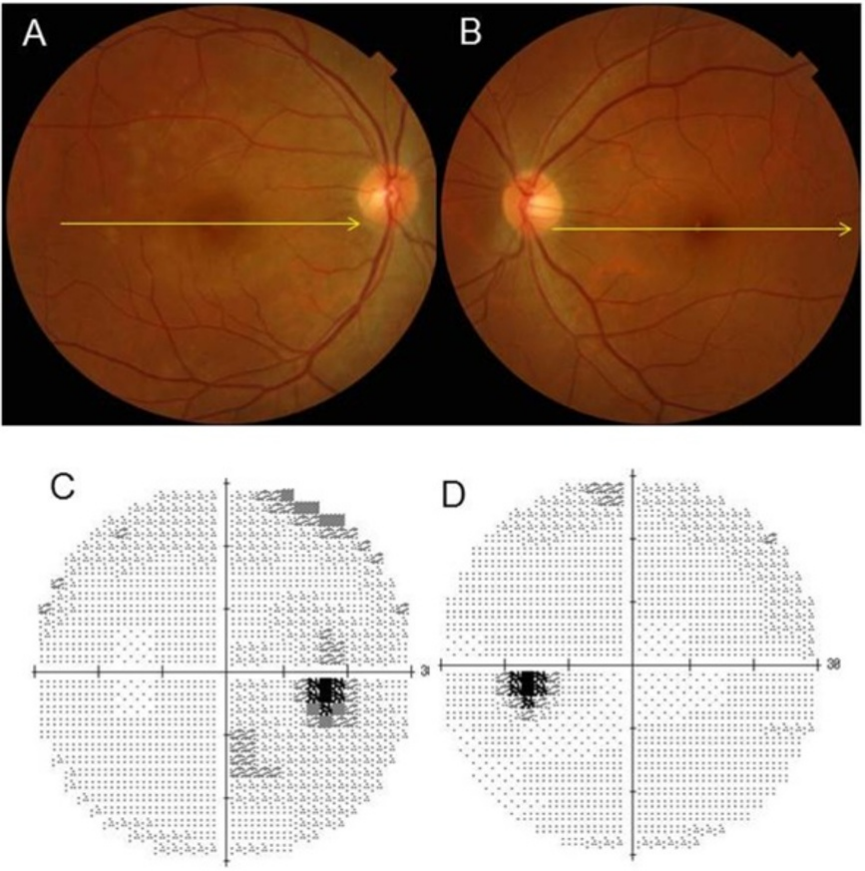
**Representative clinical findings for patient 2.** The chief complaint for this 41-year-old woman was impaired vision in her right eye. **(A) (B)** A photograph of her right fundus showed the presence of yellow dots, left eye was not affected. **(C) (D)** A visual field test was performed during the initial visit in right eye and left eye.

**Figure 2:**
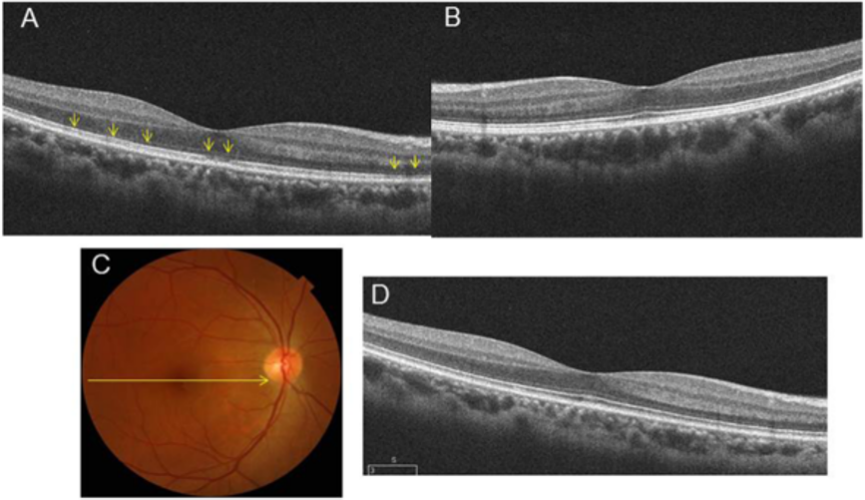
**Representative clinical findings for patient 2. (A) (B)** OCT also revealed a disrupted or irregular photoreceptor inner outer segment (IS/OS) junction in right eye indicated by the arrows, although the IS/OS junction in left eye was normal. **(C) (D)** After 4 months, the IS/OS junction in her affected eye was restored.

**Figure 3:**
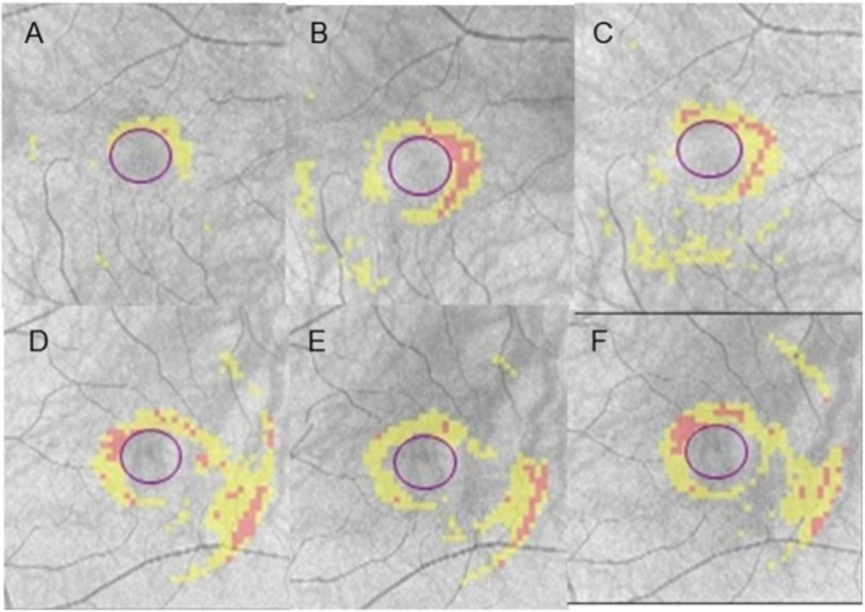
**Representative clinical findings for patient 2. (A~C)** The deviation map of the ganglion cell analysis in right eye at her initial visit, after 8 months, and 16 months. **(D~F)** The deviation map of the ganglion cell analysis in left eye at her initial visit, after 8 months, and 16 months. Signal strengths of all the Cirrus OCT measurements were 10/10.

The chief complaint for this 41-year-old woman was impaired vision in her right eye. A photograph of her right fundus showed the presence of yellow dots (Figure 1A). A visual field test during the initial visit indicated there was an enlarged blind spot in her right eye (Figure 1C). In addition, OCT also revealed a disrupted or irregular photoreceptor inner outer segment (IS/OS) junction in right eye (Figure 2A) indicated by the arrows, although the IS/OS junction in left eye was normal (Figure 2B). After 4 months, the IS/OS junction in her affected eye was restored (Figure 2D). At her initial visit, the deviation map of the ganglion cell analysis showed there was a decrease of the GCL + IPL thickness in both eyes (Figure 3A and D), even though her left eye was not affected. During the 15 months of follow-up, the deviation map did not indicate any recovery of the decreased GCL + IPL thickness. Signal strengths of all the Cirrus OCT measurements were 10/10.

### Case 2

**Figure 4:**
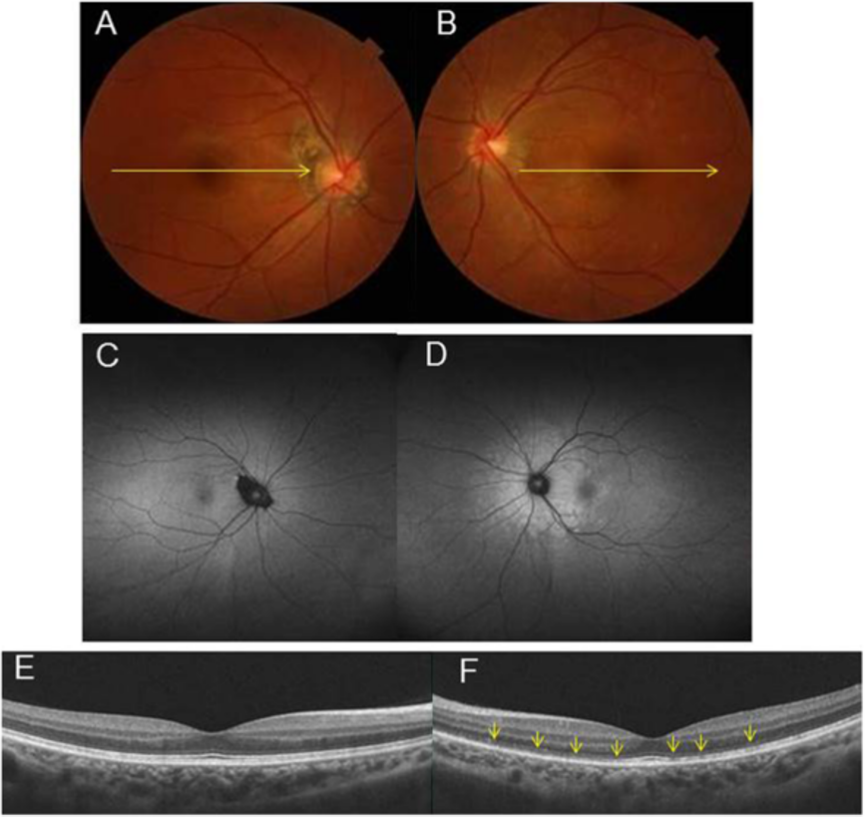
**Representative clinical findings for patient 5.** The chief complaint for this 29-year-old woman was impaired vision in her left eye. **(A) (B)** Fundus photographs. At the initial visit for this patient, a photograph of left fundus showed white dots. **(C) (D)** Fundus autautofluorescence (FAF) of Optos. FAF showed hyperautofluorescence of the area surrounding optic disc in left eye.**(E)(F)** OCT image of left eye revealed a disrupted or irregular photoreceptor inner outer segment (IS/OS) junction indicated by the arrows.

**Figure 5:**
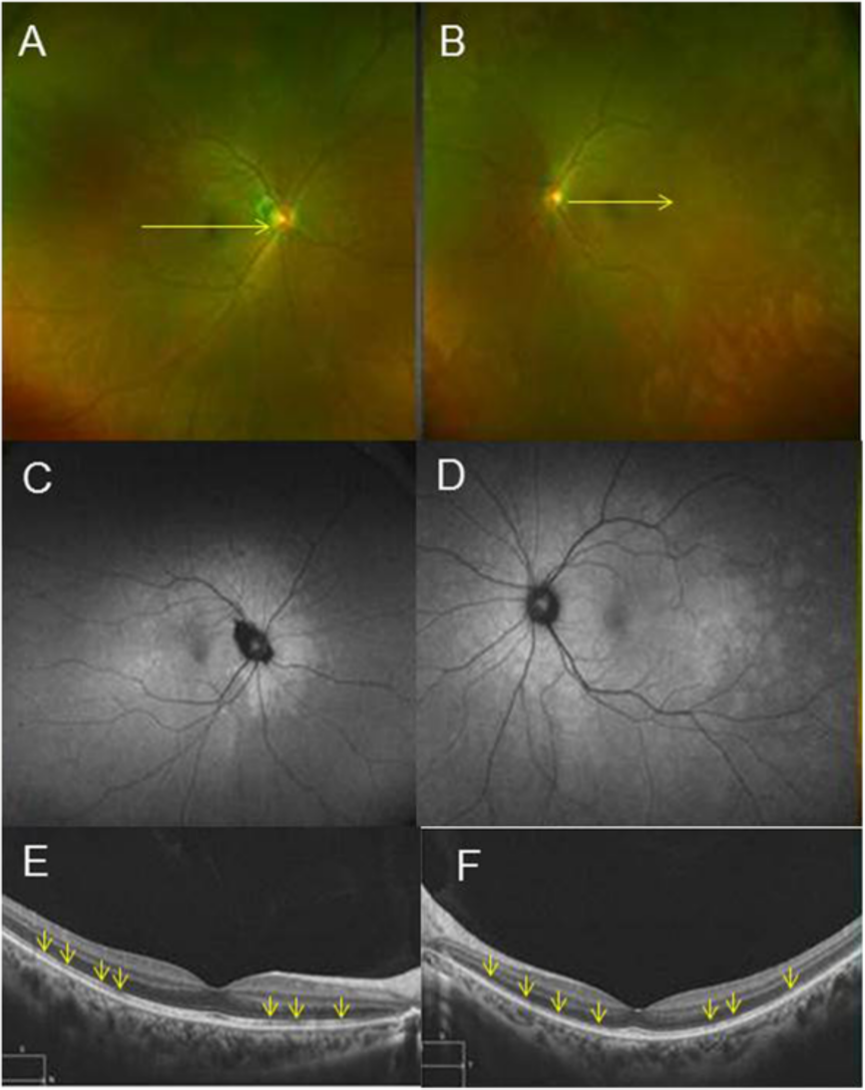
**Representative clinical findings for patient 5. (A)(B)** Fundus photographs and **(C)(D)** FAF images of Optos 1 month after her initial visit. This patient complained of metamorphopsia in right eye. FP showed that there were white dots in the posterior pole, while FAF images obtained using FAF revealed hyperfluorescence in the area surrounding the optic disc. **(E)(F)** OCT images 1 month after her initial visit. OCT showed a disrupted or irregular photoreceptor IS/OS junction in both eyes indicated by the arrows.

**Figure 6:**
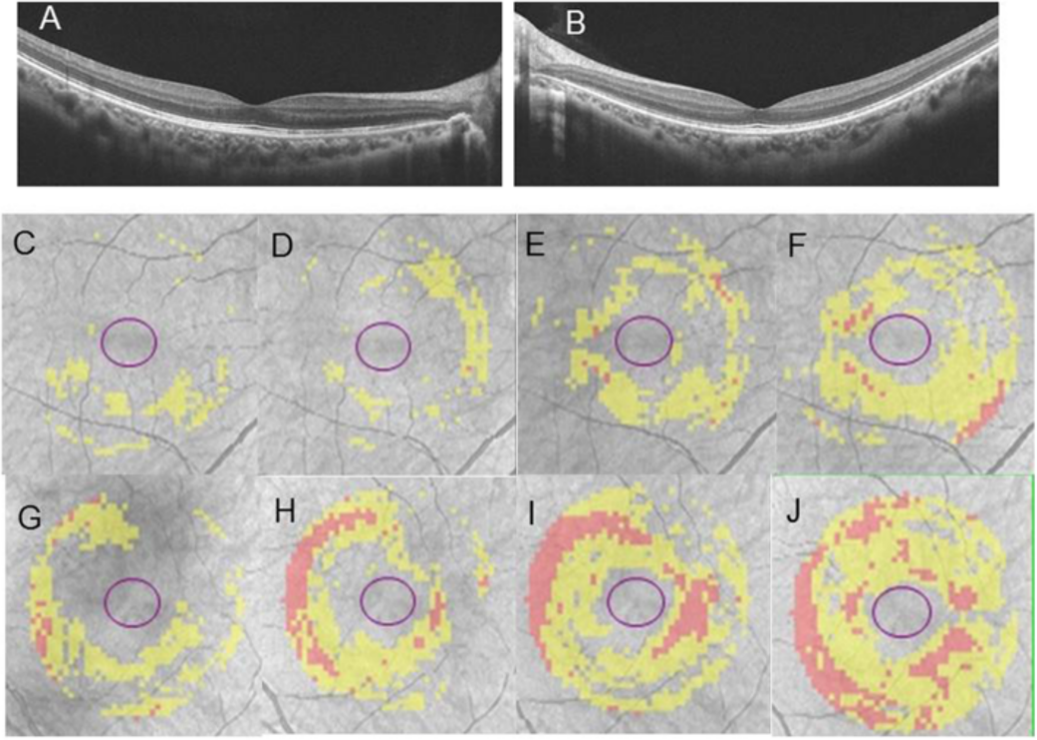
**Representative clinical findings for patient 5. (A)(B)** OCT images 4 months after initial visit. OCT indicated there was ongoing restoration of the IS/OS line in both eyes. Although the deviation map of ganglion cell analysis showed there was a decrease of the GCL + IPL thickness in right eye **(C)** and left eye **(G)** at her initial visit, her right eye was not affected. During the 4 months of follow-up (**D** and **H**; 1 month after initial visit, **E** and **I**; 2 months after initial visit, **F** and **J**; 4 months after initial visit), the deviation map did not indicate any recovery of the decreased GCL + IPL thickness.

The chief complaint for this 29-year-old woman was impaired vision in her left eye. At the initial visit for this this patient, a photograph of her left fundus showed white dots (Figure 4B) with hyperautofluorescence on FAF (Figure 4D), while OCT revealed a disrupted or irregular photoreceptor inner outer segment (IS/OS) junction (Figure 4F) indicated by the arrows. At 1 month after her initial visit, this patient complained of metamorphopsia in her right eye. FP showed that there were white dots in the posterior pole (Figure 5A), while FAF images obtained using FAF revealed hyperfluorescence in the area surrounding the optic disc (Figure 5C). OCT showed a disrupted or irregular photoreceptor IS/OS junction in both eyes (Figure 5E and F) indicated by the arrows. At 4 months after her initial visit, there was almost complete improvement of the metamorphopsia in her right and left eyes. In addition, OCT indicated there was ongoing restoration of the IS/OS line in both eyes (Figure 6A and B). Although the deviation map of ganglion cell analysis showed there was a decrease of the GCL + IPL thickness in both eyes at her initial visit, her right eye was not affected. During the 4 months of follow-up, the deviation map (Figure 6C~J) did not indicate any recovery of the decreased GCL + IPL thickness. Signal strengths of all the Cirrus OCT measurements were 10/10.

## Conclusions

Based on angiography results that show varying degrees of retinal and choroidal involvement, MEWDS is considered a chorioretinopathy [9]. Hyperautofluorescence on fundus autofluorescence (FAF) imaging also supports the hypothesis that the choroidal involvement occurs primarily in MEWDS, with secondary involvement of the RPE and the neurosensory retina [10]. However, the precise location of the initial MEWDS pathology remains unclear. Due to recent advances in OCT technology, there are now more detailed and precise quantitative assessments of retinal diseases. As a result, recent findings have shown that the lesions responsible for MEWDS appear to disrupt the photoreceptor outer segments, whose disruption can subsequently resolve [3]. Li et al. examined MEWDS and reported that the OCT and electroretinogram (ERG) findings showed unilateral ocular disease, while the visual fields and indocyanine green angiography indicated bilateral disease [3].

In our current study, 9 consecutive patients, who were diagnosed with MEWDS at Gunma University between May 2011 and June 2013, were investigated. Table 1 shows the patients’ demographic characteristics.The GCA showed there was a decrease (<1% of distribution of normals) of the GCL + IPL thickness in both the affected and fellow eyes in 7 of 9 patients. As the mean age of our 9 patients was 28.2 years and the signal strength of the OCT measurement was 10/10 in each case, we believe the current data to be reliable. This is the first report to present evidence that the inner retina is affected in both eyes, and thus, these findings might explain why visual field abnormalities are noted in both the affected and fellow eye of MEWDS patients.

**Table 1:** **Patient characteristics, best-corrected visual acuity and optical coherence tomography findings**

	Age	Gender	Eye	BCVA at initial visit	Refractive error (D)	Chief complaint	IS/OS at initial visit	Changes of GCA Deviation map in affected eye at initial visit	Changes of GCA Deviation map in fellow eye at initial visit	Final BCVA	Final condition of IS/OS	Final condition of GCA
1	37	F	L	40/50	-2.0	blurred vision	irregular	+	+	20/20	recovered	→
2	41	F	L	20/25	-0.5	blurred vision	irregular	+	+	20/16	recovered	→
3	30	F	R	20/16	-4.0	Metamorphopsia	irregular	+	prothese	20/16	recovered	→
4	16	F	B	20/16	0	Metamorphopsia	irregular	+	+	20/16	recovered	→
5	29	F	B	20/16	-3.0	Metamorphopsia	irregular	+	+	20/16	recovered	→
6	39	F	L	20/63	-1.25	Metamorphopsia	irregular	-	-	20/20	recovered	-
7	16	F	L	20/16	-4.75	Metamorphopsia	irregular	+	+	20/16	recovered	→
8	18	F	R	20/16	-1.75	Metamorphopsia	irregular	+	+	20/16	recovered	→
9	15	M	L	20/20	-1.25	blurred vision	irregular	+	+	20/16	recovered	→

M = male; F = female; R = right; L = left; B = both; BCVA = best-corrected visual acuity; D = diopter; IS/OS = photoreceptor inner/outer segment; GCA = ganglion cell analysis.

Based on the evidence that hyperautofluorescence of the fundus lesion using FAF of optos arises from peripapillary and spreads to the periphery, Inflammatory cells may derive from arachnoid space and infiltrate to retina. We would like to raise the possibility that the secondary disorder of outer segment of retina might coincide with an onset of visual symptom such as blurred vision or metamorphopsia. If the MEWDS mechanism is associated with the inflammation in arachnoid space, it could be possible that we can account for bilateral changes of inner retina. In order to definitively determine the mechanism of MEWDS, further investigations will need to be undertaken.

## Consent section

Written informed consent was obtained from the patients for publication of these Case reports and any accompanying images. All 9 of these patients consented for their details to be published. A copy of the written consent is available for review by the Editor of this journal.

## Abbreviations

MEWDS: Multiple evanescent white dot syndrome
GCA: Ganglion cell analysis
GCL: Ganglion cell layer
IPL: Inner plexiform layer
OCT: Optical coherence tomography
GCIPL: Ganglion cell-inner plexiform layer
IS/OS: Irregular photoreceptor inner outer segment
FAF: Fundus autofluorescence
ERG: Electroretinogram.
